# Case of Angina Pectoris

**Published:** 1821-11

**Authors:** Miles Marley

**Affiliations:** Member of the Royal College of Surgeons.


### *Case of Angina Pectoris.* By Miles Marley, Member of the Royal College of Surgeons.

IN the month of March last, I was desired to see the subject of the present case, Sarah Williams, residing in the neighbourhood of Fitzroy-square, about forty years of age, who had suffered from time to time, for the last eight or ten years, severe pains in her limbs; originating, I am inclined to think, from the too frequent and indiscriminate use of mercury. She was of a full, though irritable, habit; of low stature, and pale colour. Said she had experienced, occasionally, for the last three years, and frequently of late, a sense of weight and painful uneasiness in the region of the heart, which occurred after the most trifling exertion of body or mind, accompanied by great difficulty of breathing and quick successive palpitations of the heart. The exertion of walking up stairs always occasioned a paroxysm of particular violence. Her pulse was generally weak and tremulous. From the symptoms just enumerated, I had little difficulty in deciding upon the nature of the case.

Under these circumstances, I desired her to avoid every thing likely to agitate her mind; I likewise advised great regularity and temperance in her mode of living, and to pay particular attention to the regular evacuation of her bowels. I desired to be sent for, upon the least appearance of a paroxysm, to bleed her. I prescribed for her a bitter tonic draught, to be taken twice a-day. Under the above treatment, she rapidly improved. On the 24th of June, however, while attempting to walk up stairs, she fell down, and expired instantly.

*Examination.*—Upon opening into the cavity of the thorax, we noticed nothing particular; but, upon cutting into the pericardium, we found it preternaturally thickened, and void of its usual shining or glossy appearance: it did not contain more serum than is usually found in a state of health. The heart was remarkably small, of a pale colour, and very soft. The right side was gorged with blood; the left ventricle was very thin, and of a livid colour; and all the valves were more or less ossified; in which condition were also found the aorta and the two coronary arteries, particularly the latter. The viscera of the abdomen were healthy. The uterus descended lower in the vagina than it is usual in a state of health; the os tincæ was thickened, and very hard. The brain was firm and perfectly sound, with the exception of the plexus choroides, which was found loaded with blood.

This case shows us the alarming nature of the disease, and is profitable and interesting, as far as the alleviation of the symptoms go; for we must see the impossibility of doing more than palliating the effects of such malady, except in its early stage, when it may probably be in our power to check its progress. We also learn the great necessity of guarding against all sudden emotions of the mind, as well as violent bodily exertion. We likewise see the necessity of paying attention to the mode of living, carefully avoiding every thing predisposing to irritability.

*Vigo-lane, Burlington-Gardens; Sept. 6th,* 1821.

